# Comparison of the effectiveness of 0.5% and 0.25% proparacaine hydrochloride eye drops as topical anesthetics in routine ocular investigations and procedures

**DOI:** 10.22336/rjo.2024.47

**Published:** 2024

**Authors:** Rajesh Subhash Joshi

**Affiliations:** Department of Ophthalmology, Government Medical College, Maharashtra, India

**Keywords:** ophthalmic procedures, ophthalmic anesthesia, proparacaine hydrochloride drops

## Abstract

**Aim:**

To compare the effectiveness of 0.5% and 0.25% proparacaine eye drops in providing topical anesthesia for routine ocular procedures.

**Methodology:**

137 patients (274 eyes) were included in this study. They were categorized into two groups. Group A patients received 0.5% and Group B received 0.25% proparacaine drops. A single surgeon performed all the procedures. Intraprocedural and postprocedural pain scores, surgeon comfort, supplemental anesthesia, and vital parameters, were noted.

**Results:**

The mean age of the participants was 69.42 years (±12.05). An equal number of procedures were performed (n = 30) for applanation tonometry, lacrimal sac syringing, and A-scan biometry. The other procedures performed were removal of the conjunctiva (n = 5) and corneal foreign bodies (n = 16 per group), corneal scraping (n = 8 per group), and corneal suture removal (n = 18 per group).

The mean visual analog pain score during the procedure was 6.9663 in Group A and 8.0803 in Group B (*P* = 0.66). The mean postprocedural pain score was not significant (*P* = 0.21). None of the patients required any additional anesthesia during or after the procedure. The average surgeon’s experience was 0.152±0.507 in Group A and 0.111±0.402 in Group B (*P* = 0.07).

In Group A, 133 of 137 patients (97.08%), and in Group B, 132 out of 137 patients (96.35%) preferred to use the same anesthetic for future procedures. None of the patients experienced a vasovagal attack or any change in vital parameters.

**Discussion:**

The present study aimed to establish equivalence between two concentrations of proparacaine hydrochloride in which false positive and false negative errors were expected. This is the inherent challenge in the equivalence and inferiority trials. To mitigate these risks, the present study was carefully designed with specific statistical methods, sample size calculation, and rigorous methodology. Nevertheless, it is crucial to recognize that the risk of false positive errors remains, and the results should be interpreted with this in mind.

**Conclusion:**

These findings indicated that 0.25% proparacaine is a viable alternative to the standard 0.5% concentration in routine ophthalmic procedures, with the potential for improved patient comfort.

## Introduction

Topical anesthetic drops offer a way to numb the eye’s surface without the potential risks of injections. Lignocaine hydrochloride, tetracaine hydrochloride, and proparacaine hydrochloride are the local anesthetics commonly used in the form of drops for short ophthalmic procedures, such as the removal of foreign bodies from the ocular surface, measuring intraocular pressure using applanation and indentation tonometry, and checking the patency of the lacrimal passage. Nowadays, even cataract surgery using the phacoemulsification technique is performed under topical anesthesia. Proparacaine hydrochloride 0.5% is a commonly used topical anesthetic in the form of eye drops for ophthalmic procedures due to its fast onset of action, relatively prolonged effect, and rare adverse effects [1]. Diluted proparacaine hydrochloride drops have been used to manage acute corneal injuries in the emergency department [2] and in patients undergoing photorefractive keratectomy to relieve pain [3]. However, there is limited research on its use in clinical practice for routine ophthalmological procedures. Therefore, we performed an observational randomized, controlled study to assess the effectiveness of 0.25% proparacaine hydrochloride drops.

## Materials and Methods

### Sample size

To determine the difference of 5% in the two groups with a power of study of 90% and an α level of 0.05, the total sample size in the two groups was 273 eyes. The total number of eyes was rounded to 274 to achieve equal division. Therefore, each group had a sample size of 137 eyes.

A threshold of 5% was established to maintain a balance between sensitivity and specificity during our statistical analysis. This allowed the detection of meaningful differences without compromising the statistical significance. A higher threshold would have made it difficult to identify differences, while a lower threshold might have led to identifying insignificant differences as significant.

### Patient selection and study design

This study adhered to the tenets of the Declaration of Helsinki and was approved by the hospital’s medical ethics committee. The study was conducted between April 2023 and July 2023. Written informed consent was obtained from all participants.

The patients were categorized into two groups. Group A included 137 eyes, in which 0.5% proparacaine hydrochloride was used, and Group B included 137 eyes, in which a diluted form of proparacaine hydrochloride at 0.25% concentration was used. The dilution was performed by adding an equal amount of balanced salt solution (BSS) to routinely used 0.5% proparacaine hydrochloride (Paracaine, Sunways Pvt. Ltd. India). One milliliter of the diluted solution was taken, and an equal amount of BSS was added. The eyes were randomized to receive either 0.5% or 0.25% proparacaine hydrochloride. A randomization schedule was generated, and the 274 eyes were randomly assigned to either of the two groups using Research Randomizer, an online tool (https://www.randomizer.org). We recruited patients who met the predefined inclusion criteria for the study. These criteria were based on the nature of the ophthalmic procedures being performed and the patient population we aimed to assess. The study included patients who presented to the ophthalmology outpatient department of a tertiary eye care center for regular eye checkups. All patients received a routine ophthalmic evaluation. We used a secure randomization system to assign patients to the two treatment groups. This system was designed to ensure unbiased and random allocation of patients. The online randomization system was set up to allocate patients to treatment groups randomly, minimizing the risk of selection bias. In total, 137 patients (274 eyes) were numbered and randomized to one of the two groups. An independent party, not directly involved in patient care or data collection, performed the randomization process. The following procedures were performed in the two groups: measurement of intraocular pressure using a hand-held applanation tonometer, syringing of the lacrimal passage, removal of corneal and conjunctival foreign bodies, A-scan biometry for intraocular lens power calculation, corneal scraping, and corneal suture removal. One-eyed and apprehensive patients as well as those scheduled for cataract, glaucoma, pterygium, and retinal surgeries were excluded from the study. The procedure was explained to all participants and the consent was obtained. A drop of proparacaine hydrochloride was placed in the lower conjunctival fornix to anesthetize the eye by pulling down the lower eyelid. Residents performed the instillation of the drops. A single surgeon unaware of the study’s objectives conducted all procedures. After administering the drops, there was a waiting period of 3-5 minutes, which allowed for the desired effect to take place before continuing with the procedure. After the completion of the procedure, a standard 10-point visual analog scale was used to assess the intraprocedural pain [4]. Postoperative pain grading was performed 30 min. after the completion of the procedure. A trained resident graded the pain and the surgeon was absent during the pain score assessment. The patients were asked whether they would opt for a similar type of anesthetic for the same procedure in the future. The surgeon’s experience performing the procedures was also graded (Grade 0 = no discomfort, 1 = mild discomfort, 2 = moderate discomfort, 3 = patient was highly uncooperative during the procedure). Furthermore, the need for supplemental anesthesia was noted. Vital parameters, such as blood pressure, pulse rate, and oxygen saturation, were indicated using a pulse oximeter during the intraoperative and postoperative periods. The observations for each procedure were handed over to the investigator in a closed envelope.

### Statistical analysis

The demographic parameters were expressed as mean, percentages, and standard deviation. The parameters were compared using the chi-squared test. All analyses were performed using SPSS version 26.0 (IBM Corp USA). The differences were considered significant at a p-value of <0.05.

## Results

A total of 274 eyes from 274 patients were included in the study. The mean age of the participants at the time of attending the outpatient department for the routine examination was 69.42 years (±12.05, range: 45-75). There were 160 women (58.4%) and 114 men (41.6%). An equal number of participants were included in the two groups (n = 137 in each group). **Table 1** depicts the number of procedures performed in the two groups. An equal number of procedures were performed (n = 30 per group) for applanation tonometry, lacrimal sac syringing, and A-scan biometry. The other procedures included the removal of conjunctival (n = 5 per group) and corneal foreign bodies (n = 16 per group), corneal scraping (n = 8 per group), and corneal suture removal (n = 18 per group).

**Table 1:** Various ocular procedures performed in the two groups

Procedure	Group A (0.5%) proparacaine	Group B(0.25%) proparacaine
Applanation tonometry	30	30
Sac syringing	30	30
A scan biometry	30	30
Conjunctival foreign body removal	5	5
Corneal foreign body removal	16	16
Corneal scraping	8	8
Corneal suture removal	18	18
Total	137	137

The intraprocedural and postprocedural visual analog pain scores are listed in **Tables 2** and **3**. Overall, the study showed no significant difference in pain scores between the two groups. The mean VAS during the procedure was 6.9663 in Group A and 8.0803 in Group B (*P* = 0.66). The mean postprocedural pain score did not differ significantly between the two groups (*P* = 0.21). None of the patients required any additional anesthesia during or after the procedure. No procedural complications were reported in either group. The average surgeon’s experience with the procedure was 0.152±0.507 in Group A and 0.111±0.402 in Group B (*P* = 0.07).

**Table 2:** Mean intraprocedural pain scores in the two groups

Procedure	Group A (0.5%) proparacaine (Range)	Group B (0.25%) Proparacaine (Range)	P-value
Applanation tonometry	1.012 (0-4)	1.123 (0-7)	0.23
Sac syringing	1.000 (0-3)	1.000 (0-4)	0.21
A scan biometry	0.124 (0-2)	1.001 (0-3)	0.11
Conjunctival foreign body removal	1.154 (0-7)	1.155 (0-8)	0.55
Corneal foreign body removal	1.222 (0-8)	1.234 (0-9)	0.43
Corneal scraping	1.2323 (0-8)	1.235 (0-9)	0.44
Corneal suture removal	1.222 (0-4)	1.3323 (0-5)	0.22
Mean VAS	6.9663	8.0803	0.66

VAS = Visual analog score

**Table 3:** Mean postprocedural pain scores in the two groups

Procedure	Group A (0.5%) proparacaine	Group B (0.25%) proparacaine	
Applanation tonometry	0.123 (0-2)	0.135 (0-3)	0.22
Sac syringing	0.001 (0-1)	0.001 (0-1)	0.20
A scan biometry	0.000 (0-1)	0.001 (0-1)	0.10
Conjunctival foreign body removal	1.000 (0-4)	1.100 (0-4)	0.51
Corneal foreign body removal	1.001 (0-3)	1.100 (0-3)	0.40
Corneal scraping	1.200 (0-2)	1.111 (0-2)	0.20
Corneal suture removal	0.001 (0-1)	0.001 (0-1)	0.22
Mean VAS	3.326	3.449	0.21

VAS = Visual analog score

In Group A, 133 of the 137 patients (97.08%) preferred to use the same anesthetic technique for future procedures. However, two patients, each in the corneal scraping and corneal foreign body removal procedures, did not agree to use the same anesthetic. In Group B, 132 of the 137 patients (96.35%) preferred to use the same method in the future. Nonetheless, two patients in the corneal scraping and corneal foreign body removal procedures and one patient in the corneal suture removal procedure did not agree to use the same type of anesthesia. None of the patients experienced a vasovagal attack or any change in vital parameters.

## Discussion

Our findings indicated that using 0.25% proparacaine hydrochloride provided sufficient comfort for patients and surgeons during investigative and therapeutic procedures. The results showed no significant differences in pain scores during the procedures between the two groups. The VAS score was higher in Group B (8.0803), in which 0.5% proparacaine was used, compared with Group A (6.9663) (*P* = 0.66). However, the postprocedural VAS was almost equal in the two groups, meaning *P* = 0.21. Thus, our results could not be compared with those from other studies, as no studies in the literature compare the use of 0.5% and 0.25% proparacaine in various investigative procedures. Ball et al. used diluted proparacaine for acute corneal injury management in the emergency department [2]. They compared placebo with 0.05% proparacaine hydrochloride drops in patients with acute corneal injuries and concluded that proparacaine provided efficacious analgesia and did not affect wound healing. The sample size was small in their study (n = 33). In our study, we included commonly performed outpatient procedures, such as applanation tonometry, syringing of the lacrimal passage, conjunctival and corneal foreign body removal, corneal scraping, corneal suture removal, and A-scan biometry for intraocular lens power calculation. By increasing the number of procedures, this study amassed a more comprehensive and accurate dataset applicable to a broader range of patients. Our study was conducted with great care and attention to detail, considering various variables, such as age, sex, and medical history. This meticulous approach ensured that our findings were unbiased and that potential disparities were minimized. The study included 274 eyes categorized equally between the two groups. Shahinian et al. conducted a comprehensive analysis of various concentrations of proparacaine hydrochloride to evaluate their efficacy in alleviating pain following photorefractive keratectomy [3]. Their results showed that the highest concentration of the anesthetic provided the most effective pain relief but was also associated with the highest risk of side effects. Therefore, they recommended using a lower concentration for patients with a history of adverse reactions to local anesthetics. Before starting this study, we investigated the optimal protocol for instilling proparacaine hydrochloride. The protocol for instilling 0.5% proparacaine is well established. A single application of 0.5% is sufficient when applied 2-3 min before the surgery [5]. However, the protocol for diluted proparacaine use is yet to be standardized. In a preliminary investigation, five participants received diluted proparacaine hydrochloride drops (0.25%) three times at 3-minute intervals, followed by two instillations and afterward one per day. The study examined the corneal sensations and comfort levels during various procedures. The results demonstrated that a single dose of 0.25% proparacaine hydrochloride provided adequate anesthesia for most procedures.

In our study, the postprocedural VAS scores were similar between Group A and Group B (3.326 and 3.449, respectively), and the difference was insignificant (*P* = 0.21). Moreover, the surgeon’s experience with the procedure did not vary significantly between the groups, with Group A averaging 0.152±0.507 and Group B averaging 0.111±0.402 (*P* = 0.07). Also, the safety of 0.25% proparacaine hydrochloride eye drops was confirmed as none of the patients required additional anesthesia during the procedure. Most patients in both groups (Group A = 97.08% and Group B = 96.35%) preferred to use the same anesthetic for future procedures.

This study had certain limitations. First, although the sample size was adequate for the two groups, each procedure drawing a suitable sample size was beyond the scope of the study. This might have affected the generalizability of the results. A larger sample size for each procedure would have provided more robust findings. Such studies should be conducted in the future. Second, this study was performed in a single center, which might have limited the diversity of the participant population and potential variations in practice settings, thereby affecting the external validity of the findings. Third, the study was focused on specific ophthalmic procedures, and the results might not apply to all types of procedures. Generalizing the findings to other procedures would require additional research. Fourth, all procedures were performed by a single surgeon, which might have limited the expertise and experience compared to different surgeons. Fifth, this study was focused on short-term outcomes, such as pain relief and immediate safety measures, without considering potential long-term effects or patient-reported outcomes over an extended period. Performing ocular surface changes would have added safety for using low concentrations of proparacaine hydrochloride drops.

The present study aimed to establish equivalence between two concentrations of proparacaine hydrochloride in which false positive and negative errors are expected. This is the inherent challenge in equivalence and inferiority trials. To mitigate this risk, we carefully designed the present study with specific statistical methods, sample size calculation, and rigorous methodology, Nevertheless, it is crucial to recognize that the risk of false positive errors remains, and the results should be interpreted considering all these.

Considering this limitation, we emphasized the importance of replication and further research to confirm the findings of this study. Future studies with larger sample sizes and diverse patient populations are warranted to validate our conclusions.

It is important to study how the results might differ based on whether the patient has prior experience with drugs or not. However, comparing the two groups directly might be affected by factors besides drug concentrations and was beyond the scope of the study.

## Conclusion

In this randomized, controlled study comparing 0.25% proparacaine hydrochloride use with 0.5% proparacaine hydrochloride for local ophthalmic procedures, the findings suggested that both concentrations provide comparable efficacy regarding pain relief and anesthesia duration. These results support 0.25% proparacaine use as a viable alternative to the standard 0.5% concentration in routine ophthalmic procedures, with the potential for improved safety and patient comfort. However, further studies with larger sample sizes and longer follow-up periods are recommended to confirm these findings and explore additional parameters of interest. Ultimately, 0.25% proparacaine use might offer an optimized balance between efficacy and safety for local ophthalmic anesthesia.
